# High-resolution crystal structure of the human CB1 cannabinoid receptor

**DOI:** 10.1038/nature20613

**Published:** 2016-11-16

**Authors:** Zhenhua Shao, Jie Yin, Karen Chapman, Magdalena Grzemska, Lindsay Clark, Junmei Wang, Daniel M. Rosenbaum

**Affiliations:** 1 Department of Biophysics, The University of Texas Southwestern Medical Center, Dallas, Texas 75390, USA; 2 Green Center for Systems Biology, The University of Texas Southwestern Medical Center, Dallas, Texas 75390, USA

**Keywords:** cannabinoid, GPCR, crystal structure, taranabant, THC

## Abstract

The human cannabinoid G protein-coupled receptors (GPCRs) CB1 and CB2 mediate the functional responses to the endocannabinoids anandamide and 2-arachidonyl glycerol (2-AG), as well as the widely consumed plant (phyto)cannabinoid Δ^9^-tetrahydrocannabinol (THC)^1^. The cannabinoid receptors have been the targets of intensive drug discovery efforts due to the therapeutic potential of modulators for controlling pain^2^, epilepsy^3^, obesity^4^, and other maladies. While much progress has recently been made in understanding the biophysical properties of GPCRs, investigations of the molecular mechanisms of the cannabinoids and their receptors have lacked high-resolution structural data. We used GPCR engineering and lipidic cubic phase (LCP) crystallization to determine the structure of the human CB1 receptor bound to the inhibitor taranabant at 2.6 Å resolution. CB1's extracellular surface, including the highly conserved membrane-proximal amino-terminal (N-terminal) region, is distinct from other lipid-activated GPCRs and forms a critical part of the ligand binding pocket. Docking studies further demonstrate how this same pocket may accommodate the cannabinoid agonist THC. Our CB1 structure provides an atomic framework for studying cannabinoid receptor function, and will aid the design and optimization of cannabinoid system modulators for therapeutic ends.

The endocannabinoid signaling system in mammals is comprised of the endogenous lipid messengers anandamide and 2-AG and two homologous GPCRs, CB1 located in the nervous system and periphery and CB2 expressed primarily in immune cells^1^. Human CB1 and CB2 (42% sequence identity) are also activated by natural products^5^ such as THC and by synthetic cannabinoids, and can be inhibited by diverse subtype-selective and non-selective antagonists and inverse agonists^6^. CB1 is the most abundant GPCR in the central nervous system (CNS) and regulates diverse brain functions and behaviors, modulating neurotransmitter release and neuronal excitation through pre-synaptic activation of G_i/o_, GIRK channels, and arrestin/MAP kinase signaling^7^. Endocannabinoids are synthesized postsynaptically by lipases, travel across synapses in a retrograde manner^8^, and become embedded in the presynaptic membrane where they can activate CB1^9^. Beyond the CNS, CB1 signaling in peripheral tissues has been implicated in other physiological mechanisms such as release of the metabolic hormones leptin and insulin^10,11^. How lipidic or lipophilic cannabinoid agonists access their GPCR binding sites and promote receptor activation through specific binding interactions is unknown.

While THC was only discovered as the active chemical constituent of *C. sativa* in 1964^12^, humans have been consuming phytocannabinoids for their psychotropic effects for thousands of years^1^. Recently alternative therapeutic uses for cannabinoid ligands have been pursued. Because of the involvement of the endocannabinoid system in regulating energy metabolism^4^, synthetic inverse agonists such as rimonabant and taranabant proved effective in the clinic for treatment of obesity, but failed to secure regulatory approval due to adverse CNS side effects^13^. Peripheral blockade of CB1 by non-penetrant inverse agonists may represent an alternative therapeutic strategy for obesity by avoiding the CNS CB1 receptors^10^. Natural and synthetic cannabinoid ligands have also shown significant promise in treatment of neuropathic pain^2^ and epilepsy-induced seizures^3^. To gain further insight into the molecular mechanisms of cannabinoid system modulators and enable structure-based ligand design, we sought to crystallize and solve the atomic structure of the human CB1 receptor.

Obtaining diffraction-quality crystals of CB1 required optimization of both construct and purification. We carried out differential scanning fluorimetry on the detergent-solubilized receptor, which identified the inverse agonist taranabant as a ligand conferring enhanced thermostability (Methods and Extended Data Fig. 1). To promote LCP crystallization, we replaced the third intracellular loop (ICL3) of CB1 with the thermostable PGS domain, which recently proved essential in solving crystal structures of the human orexin receptors^14^. We incorporated the point mutation T210A, which was previously shown to stabilize the inactive conformation of CB1 and increase thermostability^15^. Finally, we truncated CB1^T210A^-PGS by eliminating the first 89 N-terminal residues and the C-terminus after residue 421. The engineered construct binds to the inverse agonists taranabant and rimonabant (also denoted SR141716A) nearly identically to wild-type CB1. However CB1^T210A^-PGS has a 7-fold lower affinity for the agonist CP55940, consistent with stabilization of an inactive conformation (Extended Data Fig. 2) and in agreement with the original report of the T210A mutation^15^. After purifying this construct from Sf9 insect cells (Methods and Extended Data Fig. 3), we obtained LCP microcrystals that diffracted to 2.6 Å resolution, solved the structure by molecular replacement, and refined the structure to R_free_ 0.23 (Methods and Extended Data Table 1). In the monoclinic crystals, CB1^T210A^-PGS packs in a manner such that the extracellular-facing ligand binding region is not involved in lattice contacts (Extended Data Fig. 4a), and the receptor and ligand are well-ordered with low overall B factors. While truncation of the N-terminus of CB1 was necessary to form diffraction-quality crystals, such modifications may affect the functional properties of the receptor, as indicated by the variable expression and pharmacology of tissue-specific splice variants in this region^16^. However our binding data (Extended Data Fig. 2) and previous precedent^17^ show that the basic inverse agonist and agonist binding properties of CB1 are maintained in the receptor lacking the N-terminal 89 residues (which also contains 3 consensus N-linked glycosylation motifs).

The global structure of the CB1 receptor, with its classical 7TM fold, is shown in Figure 1. By analogy to other rhodopsin family (class A) GPCRs^18^, the taranabant-bound CB1 structure represents an inactive conformation with respect to G protein binding, with a canonical ionic lock formed between R214^3.50^ and D338^6.30^ (distance 3.4 Å; Ballesteros-Weinstein numbering used in superscript). At the extracellular surface, the second extracellular loop (ECL2) and membrane-proximal N-terminal region preceding transmembrane domain 1 (TM1) form a lid over the orthosteric pocket, which almost completely shields taranabant from solvent (Fig. 1a, b). As in the structure of the lipid-activated GPCR S1P_1_, a gap between TM1 and TM7 in the extracellular leaflet (Fig. 1b) may contribute to a membrane-embedded access channel for lipophilic agonists^19^. A further dilation of the highly conserved residues I119^1.35^, F381^7.37^, and M384^7.40^ lining this channel (Extended Data Fig. 5), would be required to facilitate entry of ligands. Previous molecular dynamics simulations proposed that the endocannabinoid 2-AG enters into the homologous CB2 receptor between TM6 and TM7^20^, however these two TM domains are tightly associated in the present structure. Taranabant makes multiple contacts to both TM1 and TM7 and fills the orthosteric pocket directly inside the TM1-TM7 opening, potentially acting as a ‘plug’ to block endocannabinoid entrance. The extracellular face and lid above the orthosteric pocket contain an abundance of acidic residues, with a negatively charged surface that will energetically disfavor interaction with negatively charged ligands (Fig. 1c). This feature of CB1 may help ensure lipid binding selectivity in a bilayer containing a high concentration of negatively charged phospholipids.

The first part of the N-terminus of CB1 observed in the electron density of our crystals begins at E100. The membrane-proximal 13 amino acids preceding TM1 fold over the ligand binding pocket and interact with TM2, TM3, ECL2, and TM7 (Fig. 2a, b). This region is highly conserved in CB1 (Extended Data Fig. 6), and contributes extensively to interaction with taranabant (discussed below). The occluded nature of the CB1 orthosteric pocket was predicted by a study showing that disulfide bond formation between C98 and C107 modulates orthosteric ligand binding^21^, however this disulfide is either not present or not visible in the current structure (possibly due to cysteine capping by iodoacetamide). To assess the flexibility of the N-terminal region of CB1, we carried out a 60 nsec molecular dynamics (MD) simulation on the CB1 structure embedded in an explicit POPC bilayer, both with and without taranabant present. In both cases, the N-terminal region was highly stable over the course of the simulation, exhibiting low root mean squared deviation (rmsd) values comparable to the entire TM bundle (Extended Data Fig. 7). These results support the idea that the N-terminal region of CB1 will maintain a conformation similar to the observed structure even in the absence of ligand. Other lipid-activated GPCRs that have been structurally characterized (S1P_1_^19^ and LPA_1_^22^) contain a disulfide-crosslinked ECL2 structure that is very similar to CB1's, however these receptors' N-terminal regions are markedly different, containing α-helices that sit above the membrane and pack between ECL1 and ECL2 (Fig. 2c, d). CB1's occluded orthosteric pocket, with the N-terminal region folding over the buried hydrophobic inverse agonist taranabant, is mirrored in the structure of the visual photoreceptor rhodopsin bound to 11-cis-retinal (Fig. 2e)^23^. Further paralleling the CB1 structure, a gap between TM1 and TM7 was proposed as part of a channel for uptake and release of the lipophilic 11-cis-retinal ligand, based on the structure of the ligand-free opsin in an active conformation^24^. The opsin residues L40^1.35^, I290^7.37^, and F293^7.40^ surrounding this gap are analogous to CB1 residues I119^1.35^, F381^7.37^, and M384^7.40^ (Extended Data Fig. 5).

Taranabant is a subtype-selective inverse agonist with K_i_ = 0.13 nM for CB1 and K_i_ = 170 nM for CB2^25^. Unambiguous electron density at the orthosteric ligand binding pocket (Extended Data Fig. 4b, c) placed taranabant at an unusual site toward TM1 and TM7, which contrasts to the space occupied by inhibitors of other class A GPCRs such as the β_2_ adrenergic receptor^18^ (Fig. 3a). Taranabant adopts a conformation in which the chlorophenyl moiety extends toward TM5, the cyanophenyl buries deeper into the 7TM bundle, and the trifluoromethylpyridine projects into the putative access channel between TM1 and TM7 (Fig. 3b).

CB1's orthosteric binding pocket is highly hydrophobic, as expected for a lipid-activated receptor. Of the 24 residues within 4 Å of the ligand, there are only three polar sidechains – D104 whose acidic sidechain points toward the extracellular space, S123^1.39^ near the access channel that forms a polar contact with taranabant's trifluoromethyl group, and S383^7.39^ that has been implicated in agonist binding^26^. In contrast, a large number of hydrophobic residues (including 6 Phe, 3 Met, 2 Trp, 3 Leu, and 3 Ile sidechains) line the orthosteric pocket and make a variety of hydrophobic contacts to taranabant, burying 1109 Å^2^ of surface area (Fig. 3b). All of the taranabant contact residues on CB1 are absolutely conserved across the vertebrate lineage, except that Met can replace Ile at position 105 (Extended Data Fig. 8). The major divergence between CB1 and CB2 within the subset of binding residues lies in the membrane-proximal N-terminal region, where F102, M103, D104, I105, and F108 make van der Waals contacts to taranabant. The subtype selectivity of taranabant for CB1 may arise due to the divergence of this region between CB1 and CB2.

Taranabant (Fig. 3a) and rimonabant (Fig. 4a) have related chemical structures and similar conformational properties in isolation^27^. Docking of rimonabant into the CB1 crystal structure yielded a low-energy pose that overlaps almost completely with taranabant and contacts the same constellation of residues (Fig. 4a), supporting use of the current structure to analyze the binding modes of both ligands. Mutagenesis studies have identified several residues whose mutation caused a loss in taranabant and/or rimonabant binding affinity^27-30^. Indeed many of these residues are in contact with the ligand in the CB1 structure, including F170^2.57^, F174^2.61^, L193^3.29^, W279^5.43^, W356^6.48^, F379^7.35^, and L387^7.42^. However, several residues on TM3 and TM5 (e.g. K192^3.28^, F200^3.36^, and Y275^5.39^) are not within contact distance to taranabant, and appear to have indirect contributions to binding through structural stabilization or influences on CB1's conformational equilibrium.

To gain clues into the initial recognition of agonists by the CB1 receptor, we docked THC (a partial agonist^5^) into our crystal structure coordinates using the program Glide (see Methods). The top docking poses have the tricyclic core of THC binding between TMs 1, 2, & 7 (as intaranabant), while the C3 alkyl chain overlaps with taranabant's chlorophenyl moiety and extends toward W356^6.48^ (Fig. 4b). Conformational changes in this residue and its surroundings have been proposed as a trigger for CB1 activation, and mutation to alanine leads to enhanced stimulation (E_max_) by CB1 agonists^31^. Previous mutagenesis experiments also identified F174^2.61^, L193^3.29^, and S383^7.39^ as important residues for binding of THC or related agonists such as CP55940^26,32^. These residues are in contact or in close proximity to THC's preferred docking pose. A caveat to these calculations is that the inactive CB1 structure is not ideal for predicting high-affinity agonist interactions, although it should be noted that the crystallization construct (stabilized in an inactive conformation) still displays significant affinity for CP55940 (Extended Data Fig. 2). Finally, C355^6.47^ on the bilayer-facing side of TM6 was reported to form a covalent adduct with a THC analog containing a reactive group at the end of the C3-pentyl chain^33^. Starting with our THC pose, such crosslinking would require rotation of TM6 at the orthosteric pocket during CB1 activation and consequent disruption of packing around W356^6.48^.

While our manuscript was under review, a crystal structure of human CB1 was reported^34^ bound to the antagonist AM6538, which closely resembles rimonabant but with a nitrate group substituted on ‘Arm 2’ of the rimonabant core (i.e. the chlorophenyl moiety in Fig. 4a). While the taranabant-bound crystal structure reported here and the AM6538-bound structure are in general agreement (Extended Data Fig. 9a), there are several differences that may be important for functional interpretation and prediction. Notably, the electron density for the ligand and the important N-terminal region is weak in the AM6538-bound structure, with high B-factors in the refined model (average B = 134.3 Å^2^ for residues 99-112 and 119.5 Å^2^ for the ligand). In contrast, the equivalent region in our taranabant-bound structure is very well ordered with good density and much lower B-factors (average B = 61.7 Å^2^ for residues 100-112 and 42.0 Å^2^ for the ligand) (Extended Data Fig. 9b, c). The lack of clear density and resulting model ambiguity for the N-terminal region in the AM6538-bound structure may limit its utility for predicting the binding modes of other ligands. This is apparent in the erroneous docking prediction for taranabant, in which ‘Arm 1’ and ‘Arm 2’ (chlorophenyl and cyanophenyl groups) are swapped relative to their experimentally determined binding positions reported herein. Further biochemical and computational studies will be required to establish the relative utility of these two crystal structures as templates for ligand docking and design.

GPCRs adopt multiple conformations, creating a complex energy landscape that allows the linkage between binding of different ligands and interaction with different intracellular effectors, such as G proteins and arrestins^35^. CB1 has considerable agonist-independent constitutive activity^36^, and exhibits subtle and paradoxical pharmacological properties such as the antagonism of cannabidiol (related to THC by a bond disconnection)^5^ and inhibition by the compound ORG27569 that nonetheless allosterically increases agonist affinity^37^. Understanding these phenomena will require additional structures of CB1 in different conformational states bound to ligands (orthosteric and allosteric) of differing efficacy. Our structure of CB1 bound to taranabant represents a first step in this direction, and provides a crystallographic basis for computational design of cannabinoid system modulators.

## Methods

### Cloning, expression and purification

The wild-type human CB1 receptor gene (Uniprot Entry: P21554) was cloned into a modified pFastBac (Invitrogen) baculovirus expression vector with the haemagglutinin (HA) signal sequence followed by a Flag epitope tag at the N-terminus, and a 10×His tag at the C terminus. To facilitate receptor crystallization, the 76 N-terminal residues were removed, a TEV protease recognition site was introduced before residue K90, and the 51 C-terminal residues were deleted (truncation after P421). Residues 302-332 in the CB1 intracellular loop 3 (ICL3) were replaced with a synthetic DNA fragment containing the 196-amino acid coding sequence of *P. abysii* Glycogen Synthase (PDB: 2FBW). Finally, the mutation T210A was introduced by an adapted Multi-site Quickchange protocol (Stratagene).

The final CB1^T210A^-PGS fusion construct was transfected into DH10Bac to produce a recombinant baculovirus with the Bac-to-Bac system (Invitrogen). The recombinant baculovirus was used to infect Sf9 insect cell culture at a cell density of 2.5 ×10^6^/ml, with 1 μM taranabant (Tocris) added to the media. Infected cells were grown for 60 hours at 27°C before harvesting, and the cell pellets were stored at -80 °C for future use.

Sf9 cell membranes were disrupted by thawing frozen cell pellets in a hypotonic buffer containing 10 mM Tris pH 7.5, 1 mM EDTA, 160 μg ml^-1^ benzamidine, 100 μg ml^-1^ leupeptin, 2 mg ml^-1^ iodoacetamide and 1μM taranabant. The cell membranes were centrifuged at 10,000g for 20 min at 4 °C. Membrane pellets were solubilized in a buffer containing 50 mM HEPES, pH 7.5, 500 mM NaCl, 1% (w/v) n-dodecyl-b-D-maltopyranoside (DDM; Anatrace), 0.2% sodium cholate, 0.2% cholesteryl hemi-succinate (CHS), 10% glycerol, 160 μg ml^-1^ benzamidine, 100 μg ml^-1^ leupeptin, 2 mg ml^-1^ iodoacetamide and 10 μM taranabant for 1 hour at 4°C. The supernatant was isolated after ultra-centrifugation for 30 min at 100,000g, and incubated with Ni-NTA agarose beads (GE Healthcare) in batch for 3 hours at 4°C. After binding, the beads were collected by centrifugation at 100g and washed with five volumes of Ni-NTA wash buffer (50 mM HEPES, pH 7.5, 500 mM NaCl, 0.05% (w/v) n-dodecyl-b-D-maltopyranoside (DDM; Anatrace), 0.01% sodium cholate, 0.01% cholesteryl hemi-succinate (CHS), 10% glycerol, 50 mM imidazole, 160 μg ml^-1^ benzamidine, 100 μg ml^-1^ leupeptin and 1 μM taranabant). After transfer to a gravity column, beads were washed with 15 column volumes of Ni-NTA wash buffer, and receptor protein was eluted in Ni-NTA wash buffer with 200 mM imidazole and 2 mM calcium. The eluted protein was then loaded by gravity flow over anti-Flag M1 affinity resin. Detergent was exchanged from 0.05% DDM to 0.05% lauryl maltose neopentyl glycol (LMNG) on the M1 resin. Finally the pure receptor was eluted with 0.2 mg ml^-1^ Flag peptide and 5 mM EDTA. TEV protease (1:10 w/w) and PNGase F were added to the eluate, and protein was incubated at 4°C overnight. Finally, the receptor was run on a Superdex 200 size exclusion column (GE Healthcare) with buffer containing 20 mM HEPES, pH 7.5, 150 mM NaCl, 0.05% LMNG, and 1 μM taranabant.

### Differential Scanning Fluorimetry

Protein samples were purified and prepared in the absence of ligand (apo), with taranabant, or with rimonabant, as described above. Differential Scanning Fluorimetry (DSF) assays were performed in 96-well PCR plates using an RT-PCR machine (CFX96, Bio-Rad). Standard assay conditions (25 μL) were 25mM HEPES pH 7.5, 150 mM NaCl, 0.01% LMNG, 0.002% CHS and 10 μM corresponding ligands. The protein concentration was 2 μM and the BODIPY FL-L-cystine dye^38^ was added at 2 μM final. All reactions were incubated at 4°C for 20 min before scanning in the PCR machine. The fluorescence was measured at 0.5°C temperature intervals from 4°C to 90°C by using the FAM filter set (450–490 nm excitation, 515–530 nm emission).

### Crystallization

Purified receptor was concentrated to 55 mg ml^-1^ using a 100 kDa cut-off Vivaspin column (Sartorius), and crystallized using the lipidic cubic phase method. The concentrated receptor was reconstituted into a lipid mixture containing monoolein plus 10% (w/w) cholesterol (Sigma), in a ratio of 2:3 receptor-to-lipid (by weight). Mixing was performed at room temperature using a syringe mixing apparatus as previously described^39^. The mesophase was dispensed in 40 nl drops onto 96 – well glass plates and overlaid with 800 nl precipitant solution using a Gryphon LCP robot (Art Robbins Instruments). Crystals grew to full size after 2 weeks at 20°C in the following overlay precipitant condition: 31% PEG400, 100mM Sodium Citrate pH5.5, 100mM magnesium sulfate. The crystals were harvested from LCP setups using MiTeGen loops and cryoprotected in liquid nitrogen.

### Data collection and processing

X-ray diffraction data were collected at GM/CA-CAT beamline 23ID-B at the Advanced Photon Source (APS), Argonne National Laboratory, Argonne IL, equipped with an Eiger 16M detector. Data sets were acquired using a beam size of 20 μm with 1.033 Å wavelength X-rays. For each crystal, 50 0.4° oscillation images were collected, with 1 sec exposure and without attenuation of the beam. Due to radiation damage of crystals, a 97% complete diffraction data was merged from 42 crystals and scaled using HKL3000^40^. The dataset was processed in space group P2_1_, and a resolution cutoff of 2.6 Å was selected by examining CC_1/2_ values after anisotropy correction in HKL3000.

### Structure determination and refinement

**Figure 1:**
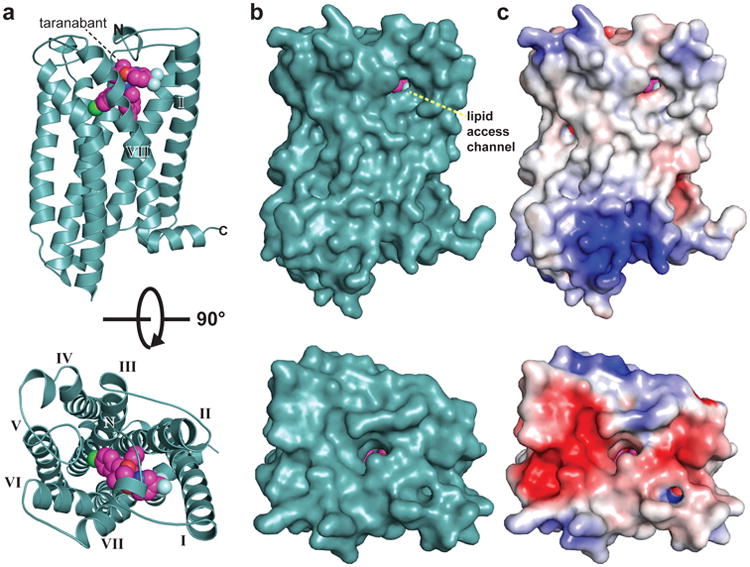
Global structure of CB1 bound to taranabant (a) CB1 is represented as a teal cartoon. The taranabant ligand is shown as spheres with magenta carbons. Views are from within the plane of the membrane (top image) and from the extracellular space (bottom image). (b) Solvent accessible surface representation of CB1 from the same views as in (a). (c) CB1 surface representation colored according to electrostatic potential.

The structure of CB1^T210A^-PGS in complex with taranabant was solved by molecular replacement with Phaser^41^ using human S1P_1_receptor^19^ (PDB: 3v2y) and PGS^42^ (PDB: 2bfw) as independent search models. The solution was improved through iterations of manual building in Coot^43^, followed by refinement using Refmac5^44^. Translation-libration-screw (TLS) refinement was used to model atomic displacement factors. Refinement parameters for the taranabant ligand were generated using the PRODRG^45^ web server. Resulting statistics for data collection and refinement are included in Extended Data Table 1. The final structure had 96.6% of residues in the favored region of the Ramachandran plot, 3.4% in the allowed region, and 0 residues disallowed. Figures were prepared using Pymol (Schrodinger LLC). The electrostatic potential surface shown in Fig. 1c was calculated using APBS^46^.

### Binding of ligands to the CB1 receptor

Ligand binding experiments on membranes containing CB1 wild-type, CB1-PGS, and CB1^T210A^-PGS were carried out based on a previously published protocol^28^. Sf9 cells expressing each construct (without any ligand present) were used to generate membranes by dounce homogenization and differential centrifugation^18^. Saturation binding was carried out by incubating 1.5-5 μg of membranes with different concentrations of [^3^H]SR141716A (54 Ci/mmol, Perkin-Elmer) between 0.05 and 25.6 nM in assay buffer (25 mM Tris pH 7.5, 5 mM MgCl2, 1 mM EDTA) containing 0.1% protease-free BSA in a final volume of 250 μl per tube. Reactions were incubated at 30°C for 1 hr, and then quenched with 250 ul assay buffer with 5% BSA. Non-specific binding was determined using reactions containing 1 μM unlabeled ligand. Reactions were separated on a vacuum manifold using GF/C filters (pre-soaked in assay buffer + 0.5% PEI) to retain membranes and discard unbound ligand. After washing 4 times with cold assay buffer, bound radioactivity was quantified using a scintillation counter. For competition binding experiments, aliquots of membranes were incubated with 3 nM [^3^H]SR141716A, and varying concentrations of competitor ligands (taranabant or CP55940) were included in the binding reactions. All binding experiments were carried out as three independent experiments, each performed in duplicate. Data analysis and fitting was performed with GraphPad Prism (GraphPad Software Inc.).

### Molecular dynamics simulations

The system used for MD simulation consisted of one copy of CB1 receptor (PGS domain removed), taranabant, 240 POPC molecules (1-palmitoyl-2-oleoyl-sn-glycero-3-phosphocholine), 48 Na^+^, 57 Cl^-^, and 17087 water molecules. MD simulations were performed with periodic boundary condition to produce isothermal-isobaric ensembles using the modified PMEMD.CUDA program in AMBER 14^47^. Temperature was regulated using Langevin dynamics^48^ with a collision frequency of 5 ps^-1^. Pressure was regulated using the isotropic position scaling algorithm with the pressure relaxation time set to 1.0 picosecond. The integration of the equations of motion was conducted at a time step of 1 femtosecond for the relaxation phase and 2 femtoseconds for the equilibrium and sampling phases. After 5-nanosec equilibration, a 55-nanosecond MD simulation was performed at 298 K, 1 bar to produce NTP (constant temperature and pressure) ensembles. The TM helices were very stable in both simulations and the mean rmsd values were 1.52±0.13 and 1.45 ± 0.23 Å for the apo and complex forms, respectively. The rmsd values of the membrane-proximal N-terminal region of the complex form (0.96 ± 0.24 Å) are smaller compared to those of the apo form (1.28 ± 0.19 Å).

### Docking of rimonabant and THC

Molecular docking was performed for taranabant, rimonabant, and THC using Glide^49,50^, implemented in the Schrodinger software package (www.schrodinger.com). Different protocols of receptor preparation, grid generation and flexible ligand docking were evaluated and the one that produced the best docking scores was adopted. The optimal Glide protocol for CB1 included: only optimize hydrogen atoms in the receptor preparation; allow hydroxyl and thiol groups of T197, S383 and C386 to be rotatable; use the standard precision scoring function. We first tested our docking protocol by re-docking the taranabant ligand from the crystal structure. The best docking scores were -12.76 and -12.59 kcal/mol for the crystal conformation and a 3D conformation generated without any initial bias using the Concord program (www.certara.com), respectively. The rmsd between the crystal structure and docking pose was 0.55 Å for the Concord conformation. Next, the antagonist rimonabant and the partial agonist THC were docked to the binding pocket using the same protocol. The docking scores of the best docking poses were -8.99 and -9.36 kcal/mol for rimonabant and THC, respectively.

## Extended Data

**Extended Data Figure 1:**
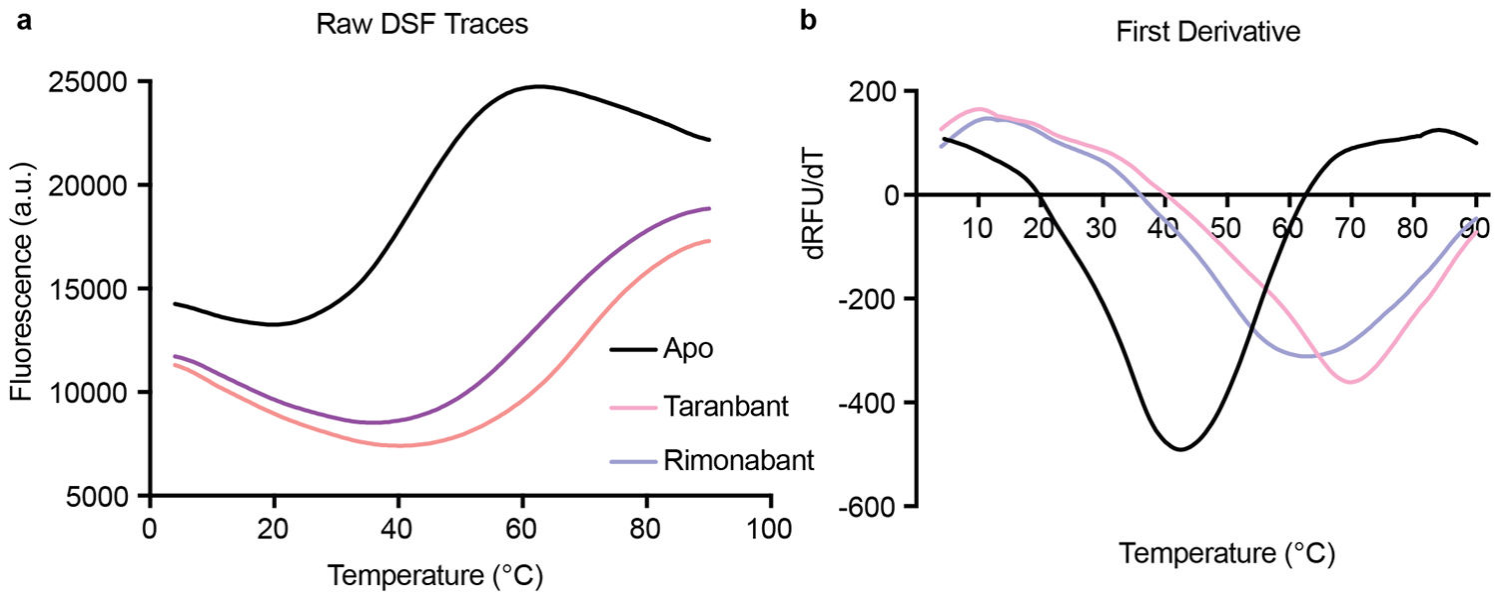
Differential scanning fluorimetry (DSF) on purified CB1-PGS (a) Raw DSF traces of the receptor in the apo state or bound to each antagonist. (b) First derivative analysis of data in (a)

**Extended Data Figure 2:**
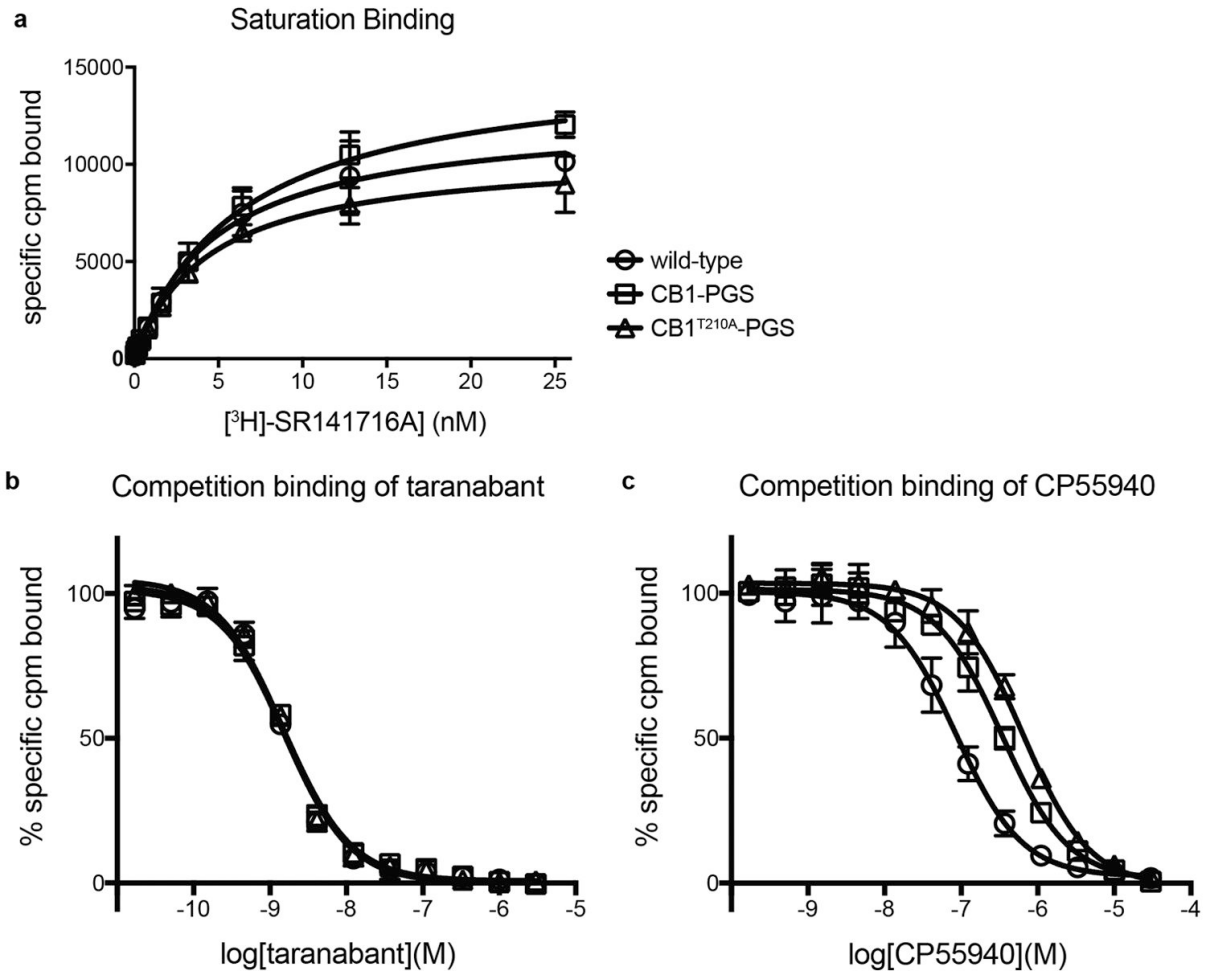
Ligand binding properties of CB1 constructs (a) Saturation binding of the antagonist ^3^H-SR141716A (tritiated rimonabant radioligand) to wild-type CB1, CB1-PGS, and CB1^T210A^-PGS. Error bars represent s.d. for three separate experiments, each performed in duplicate. The fitted K_d_ values (± s.e.) for these three constructs are 4.8 ± 0.7 nM, 6.3 ± 0.6 nM, and 4.4 ± 0.5 nM, respectively. (b) Competition binding of taranabant to the wild-type CB1 receptor, CB1-PGS, and CB1^T210A^-PGS. Error bars represent s.d. for three separate experiments, each performed in duplicate. The Ki values (± s.e.) of the three constructs for taranabant are 0.94 ± 0.17 nM, 1.10 ± 0.16 nM, and 0.91 ± 0.16 nM, respectively. (c) Competition binding of the agonist CP55940 to the wild-type CB1 receptor, CB1-PGS, and CB1^T210A^-PGS. Error bars represent s.d. for three separate experiments, each performed in duplicate. The Ki values (± s.e.) of the three constructs for CP55940 are 53 ± 12 nM, 230 ± 43 nM, and 384 ± 62 nM, respectively.

**Extended Data Figure 3:**
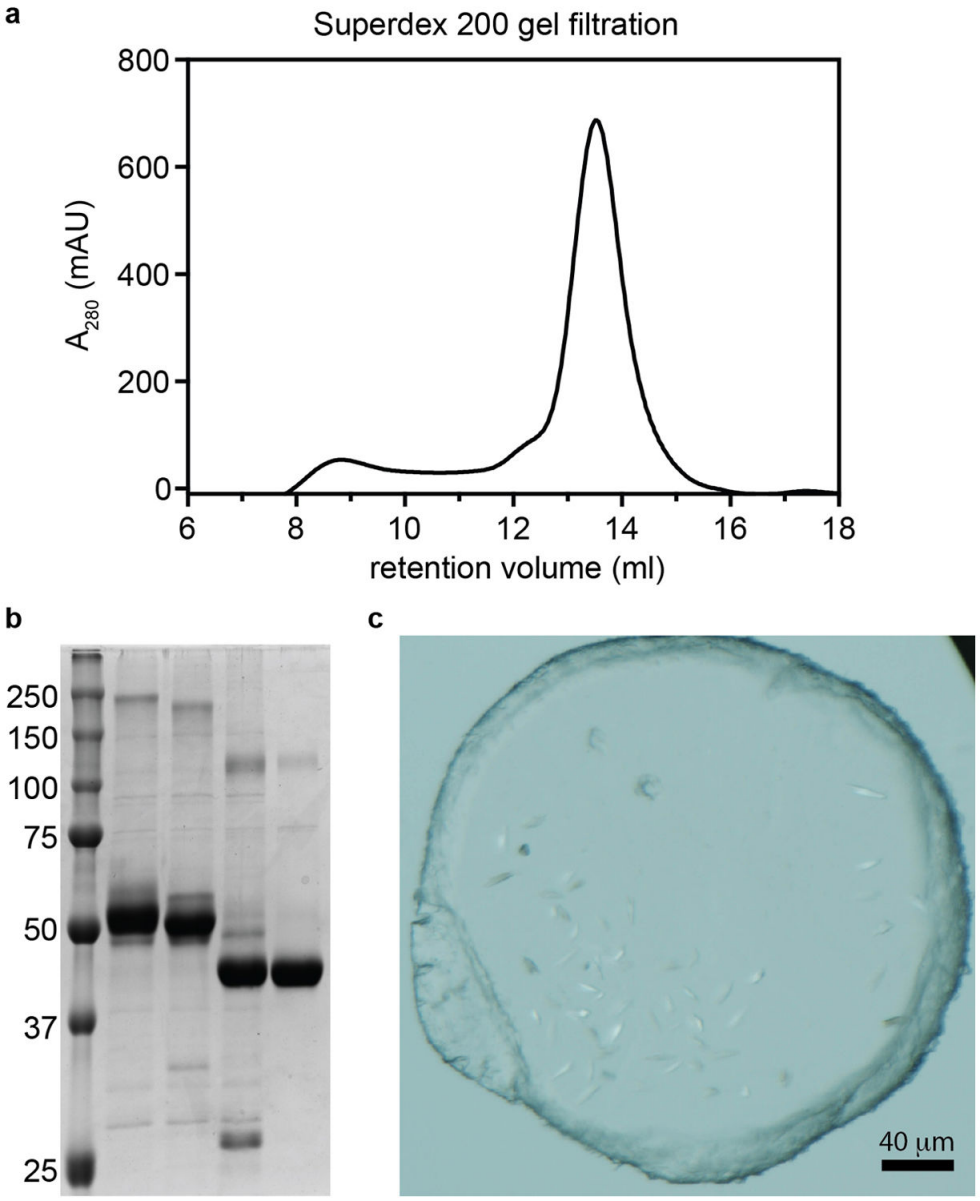
Purification and crystallization of CB1^T210A^-PGS (a) Superdex 200 gel filtration trace of receptor after Ni IMAC chromatography and M1 anti-FLAG chromatography (see Methods). (b) SDS-PAGE analysis of samples at different stages of purification. 5 lanes from left to right are: Markers (MW in kD at left); IMAC/FLAG-purified receptor; same sample after treatment with PNGaseF; receptor after TEV cleavage (removing 89 N-terminal amino acids); final sample after Superdex 200 gel filtration. (c) Light microscopy image showing LCP microcrystals of CB1^T210A^-PGS used to collect diffraction data.

**Extended Data Figure 4:**
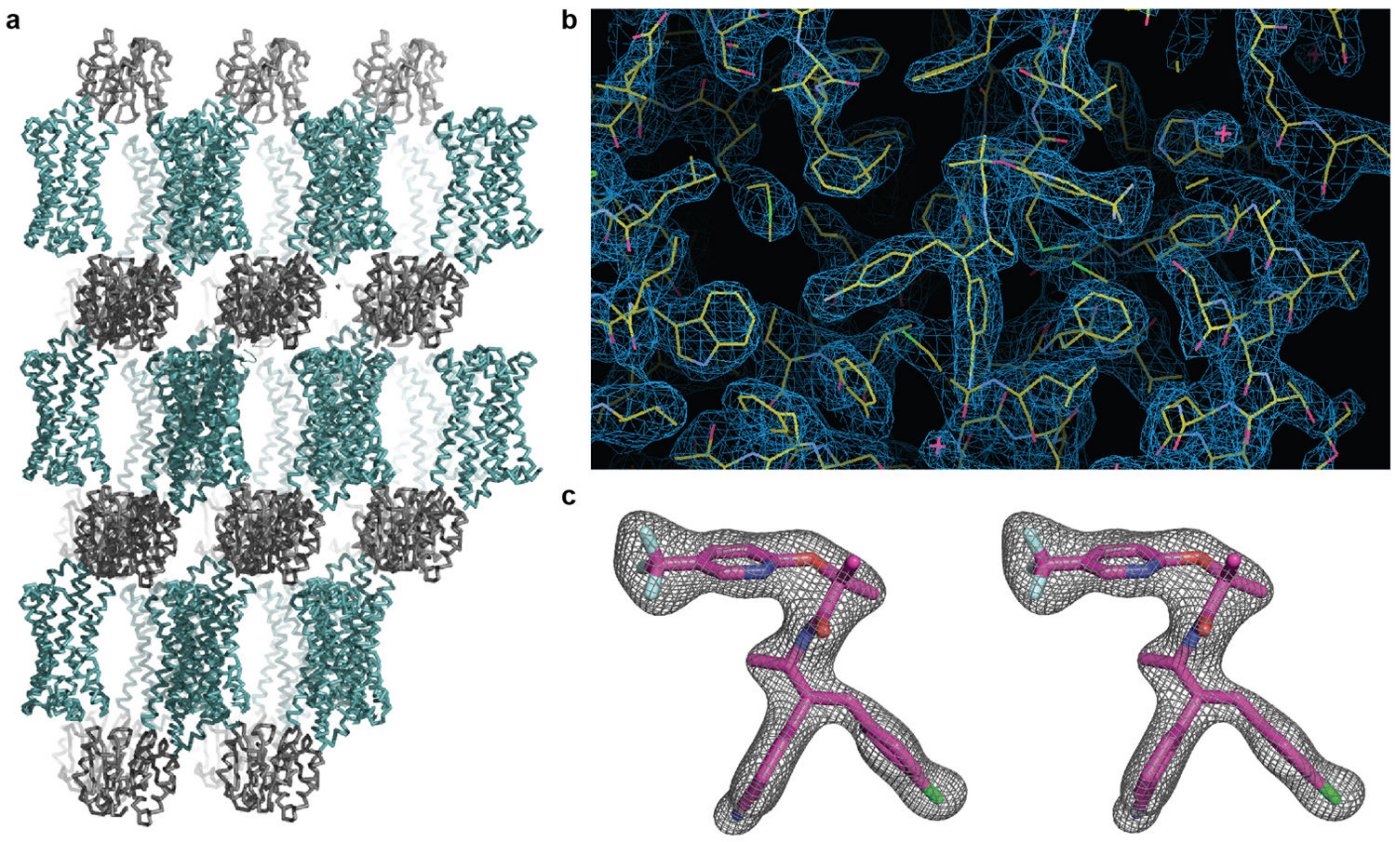
Packing and electron density in the CB1^T210A^-PGS crystals. (a) Lattice packing interactions in the monoclinic crystals of CB1^T210A^-PGS. Protomers are shown as ribbons, with the receptor component of the fusion protein colored teal and the PGS domain colored gray. (b) 2F_o_-F_c_ electron density map (contoured at 1.2 σ) of taranabant and the surrounding ligand binding residues. Protein and ligand are represented as yellow sticks. (b) Stereoview of 2F_o_-F_c_ electron density (contoured at 1.5 σ) for only the ligand taranabant (magenta sticks).

**Extended Data Figure 5:**
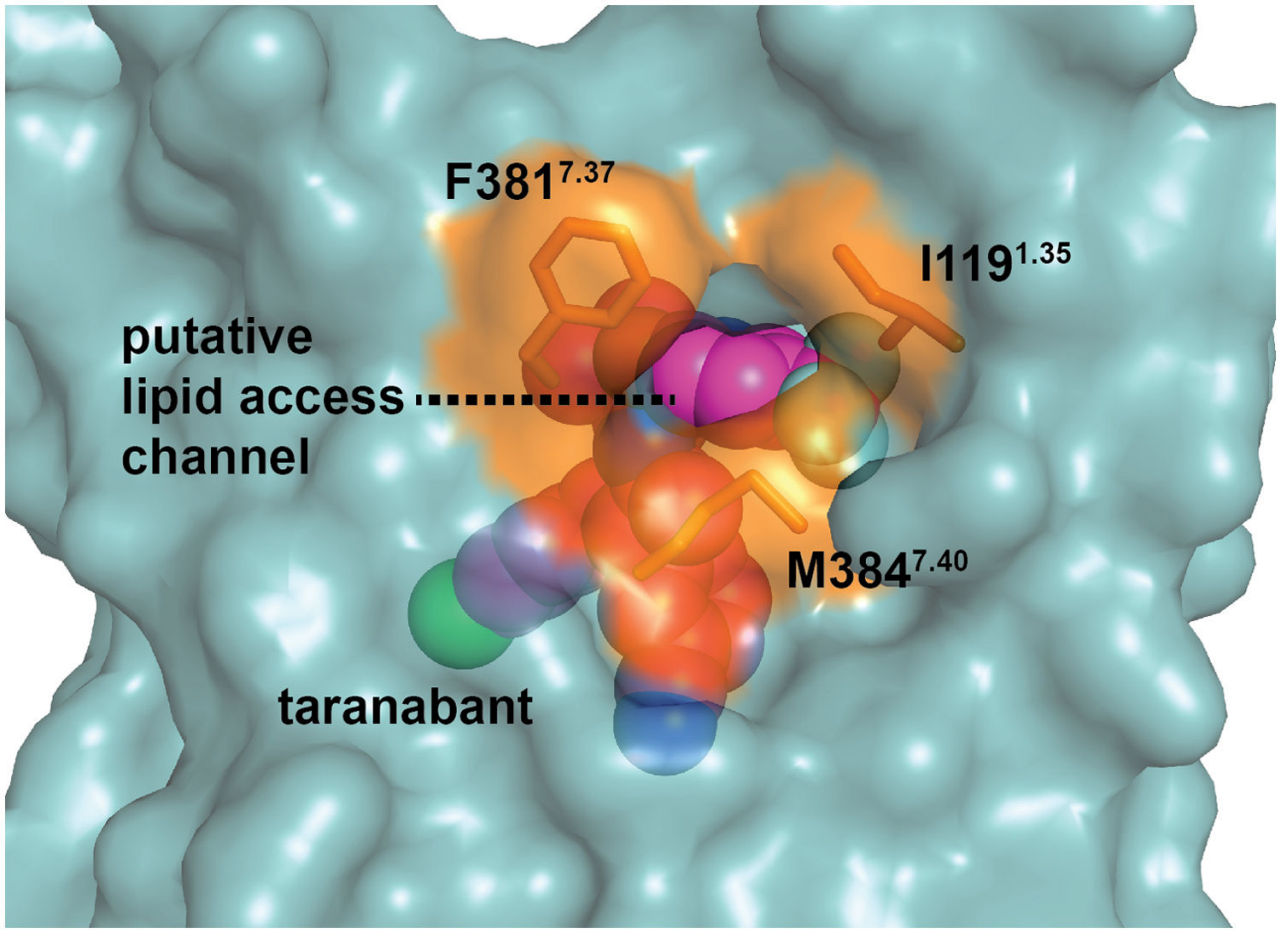
Residues lining the putative lipid access channel of CB1. The receptor is shown as a teal transparent surface, and taranabant is in magenta spheres. The three residues lining the channel are shown as orange sticks and their solvent accessible surfaces are colored orange.

**Extended Data Figure 6:**
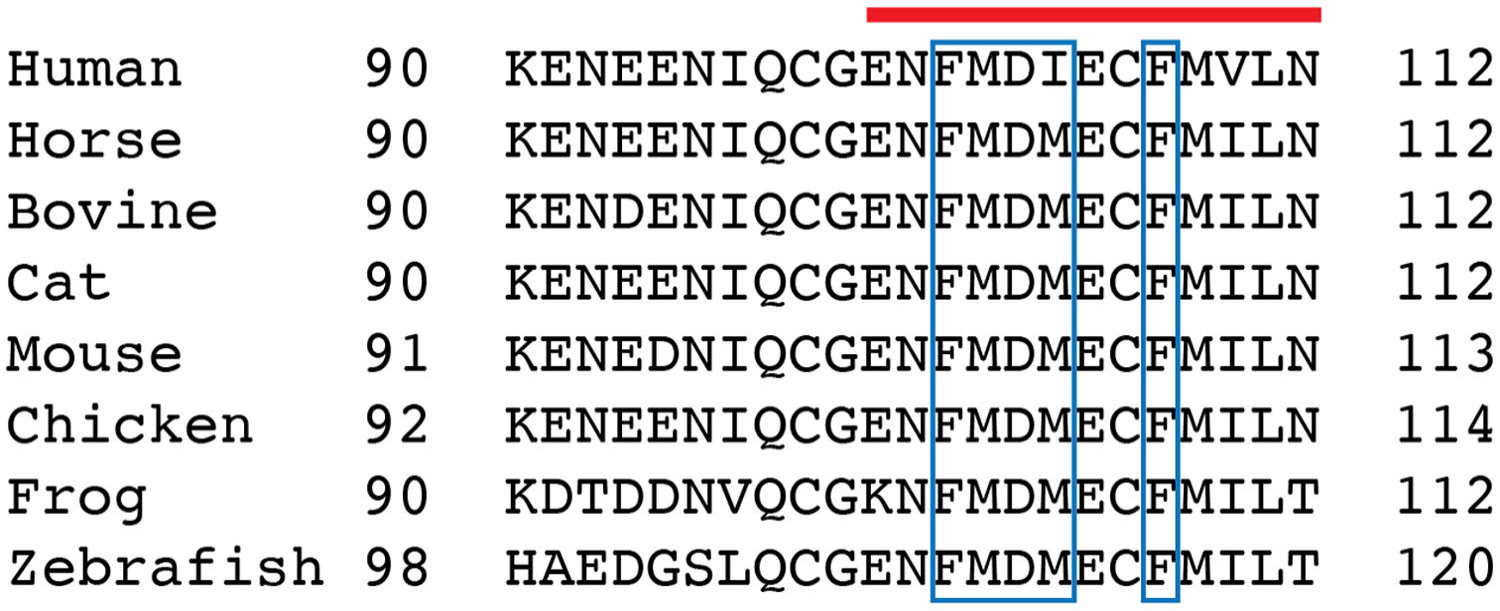
Sequence alignment of the membrane-proximal N-terminal region of CB1 from different vertebrate species. ‘Frog’ is *Xenopus laevis.* The red bar on top indicates the part of this region that is structured and visible in the electron density of the CB1 crystals. The blue box denotes positions that make contact to taranabant. Alignment was made using Clustal Omega (https://www.ebi.ac.uk/Tools/msa/clustalo/).

**Extended Data Figure 7:**
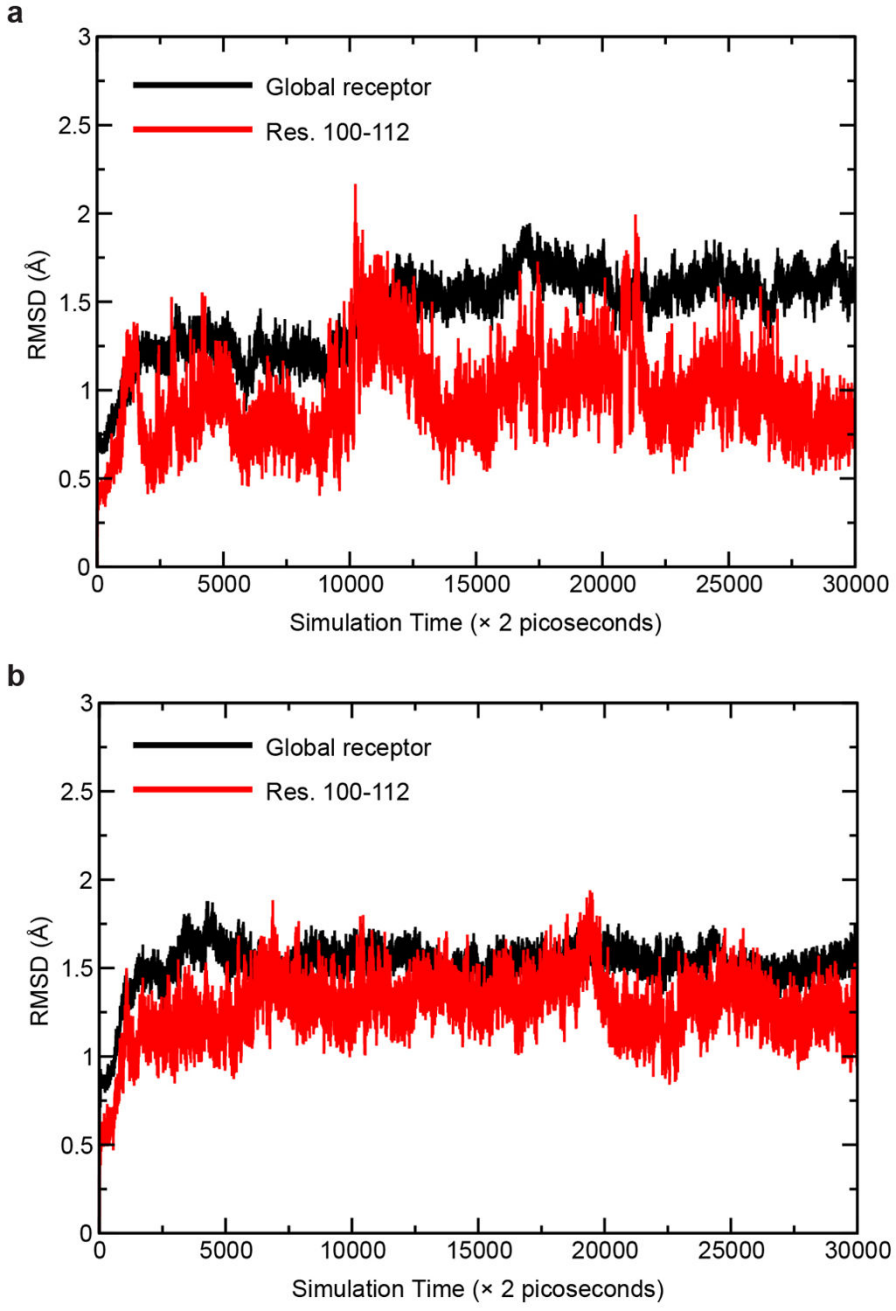
Molecular dynamics (MD) simulation of the CB1 structure (a) 60 nsec MD simulation of the CB1 receptor (after removing the PGS fusion protein) with taranabant present. Black trace is for the entire receptor, red trace is for only the structured membrane-proximal N-terminal region. (b) 60 nsec MD simulation of the CB1 receptor without a ligand present.Black trace is for the entire receptor, red trace is for only the structured membrane-proximal N-terminal region.

**Extended Data Figure 8:**
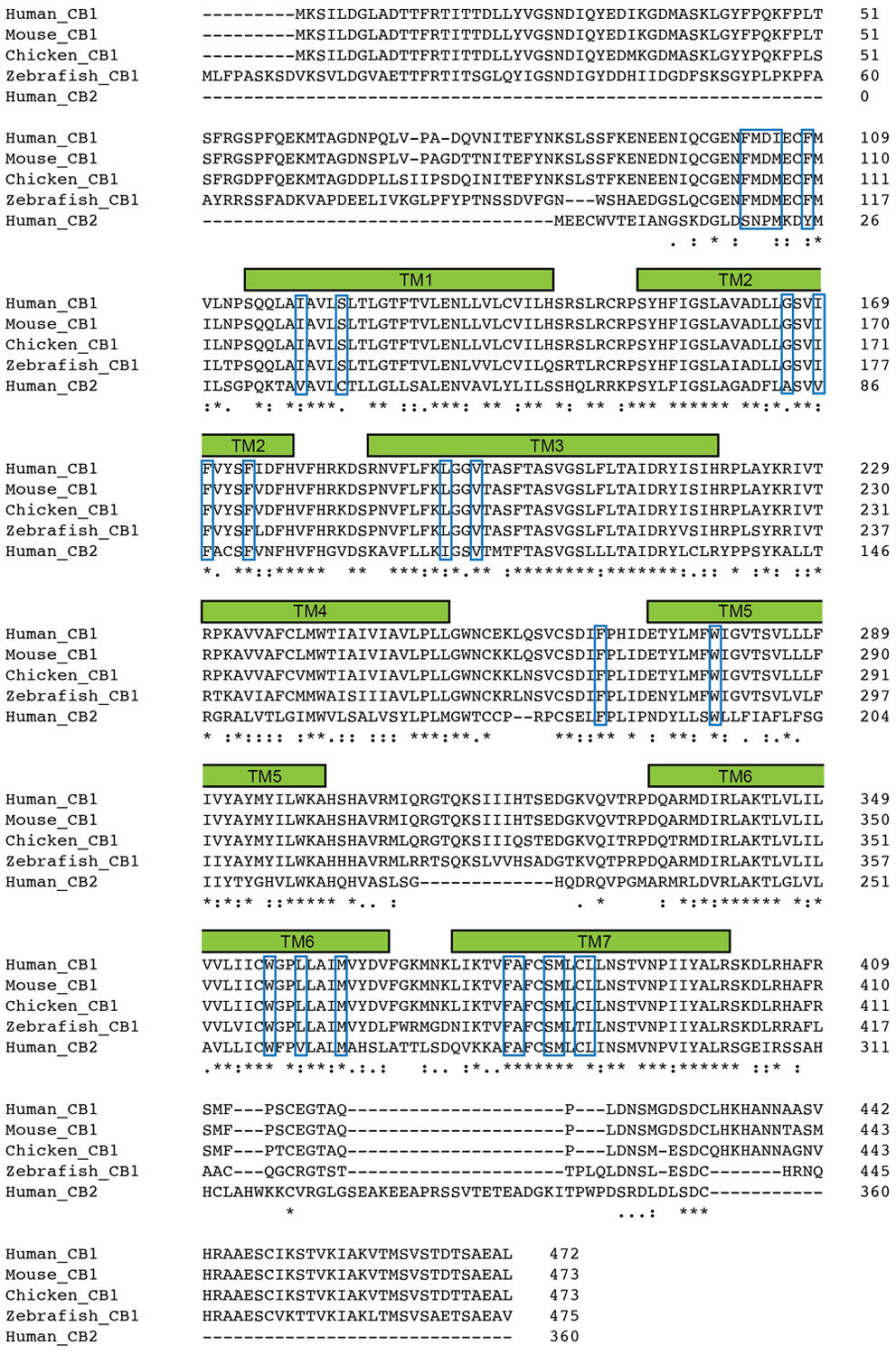
Sequence alignment of the entire sequence of CB1 from several different species, along with human CB2. The blue boxes denote positions that make contact to taranabant within a 4Å cutoff. The alignment was made using Clustal Omega (https://www.ebi.ac.uk/Tools/msa/clustalo/).

**Extended Data Figure 9:**
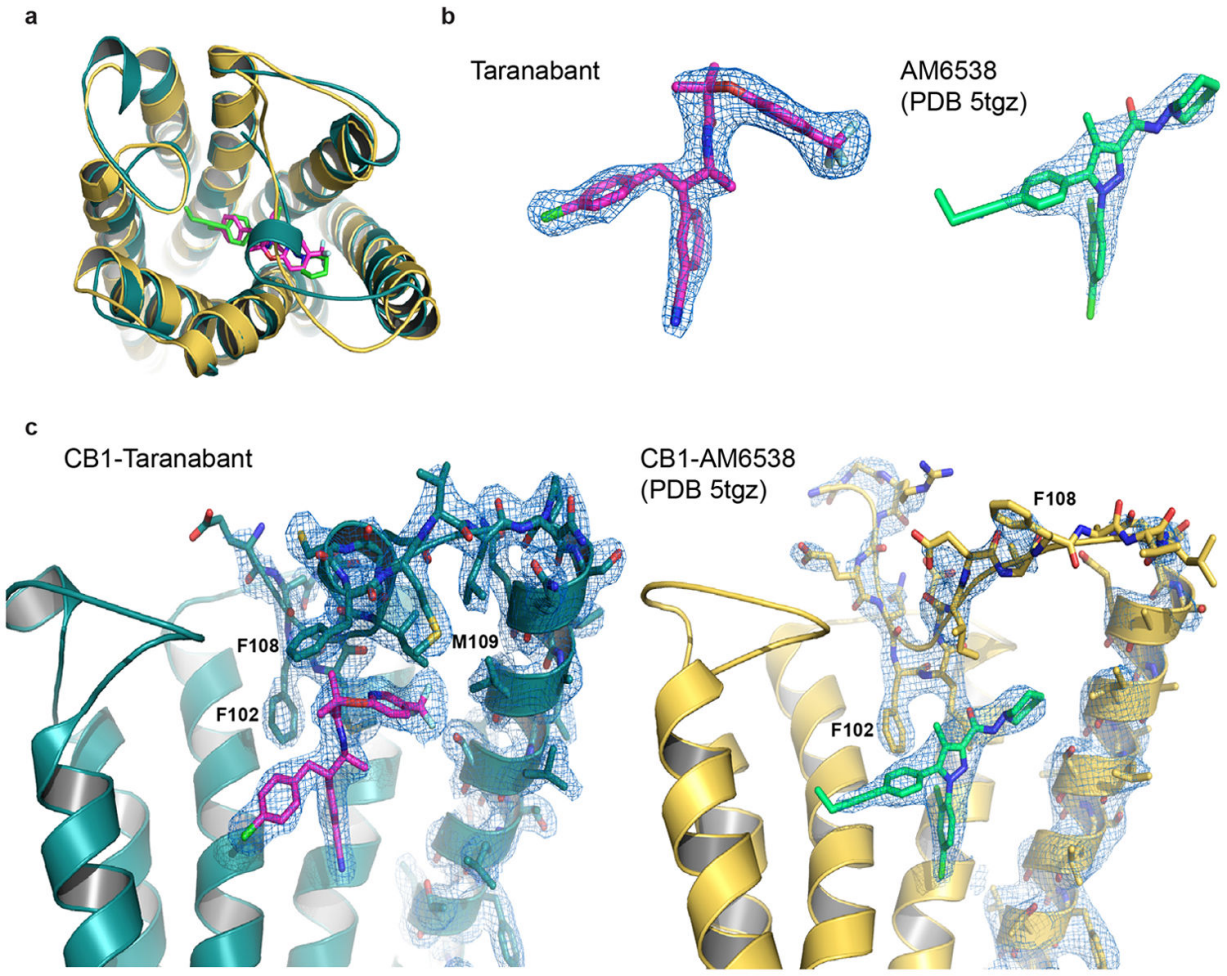
Comparison of the structures of CB1 bound to taranabant and CB1 bound to AM6538 (ref. 34, PDB 5tgz) (a) Superposition of the two CB1 structures viewed from the extracellular space. The taranabant-bound structure is shown as a teal cartoon (ligand as magenta sticks), while the AM6538-bound structure is shown as a gold cartoon (ligand as green sticks). (b) Comparison of 2F_o_-F_c_ electron density (contoured at 1.5 σ) for the ligands in each structure. At left is taranabant from the current structure, at right is AM6538 from ref. 34. (c) Comparison of the membrane-proximal N-terminal regions in each structure. At left is a side view of CB1 from the current structure, with 2F_o_-F_c_ electron density (contoured at 1.0 σ) shown for the N-terminal region, TM1, and taranabant. At right is the analogous side view of CB1 from ref. 34 (gold cartoon), with 2F_o_-F_c_ electron density (contoured at 1.0 σ) shown for the N-terminal region, TM1, and AM6538.

**Extended Data Table 1:** Data collection and refinement statistics

	CB1-PGS with Taranabant¶
**Data collection**	
Space group	P2_1_
Cell dimensions	
*a, b, c* (Å)	50.7, 80.4, 81.2
*β* (°)	91.7
Resolution (Å)	50.00-2.60 (2.69-2.60)†
*R_sym_ or R_merge_*‡	0.19 (NA)
*I/σI*	7.43 (0.96)
Completeness (%)	96.8 (96.9)
Redundancy	5.4 (5.1)
*CC*_1/2_ in highest shell	0.69
**Refinement**	
Resolution (Å)	50-2.60
No. reflections	11084
*R_work_/R_free_*	0.19/0.23
No. atoms	
Protein	3788
Ligand/ion	57
Other (Lipid and water)	124
B-factors	
Receptor	45.5
Fusion protein	38.3
Ligand	42.0
Ion	91.0
Other (Lipid and water)	44.4
R.m.s deviations	
Bond lengths (Å)	0.008
Bond angles (°)	1.20

¶ Diffraction data from 42 crystals were merged into a single data set.

† Values in parentheses are for highest-resolution shell.

‡ *R*_merge_>1 is statistically meaningless, Scalepack^40^ does not report it.

## Figures and Tables

**Figure 2:**
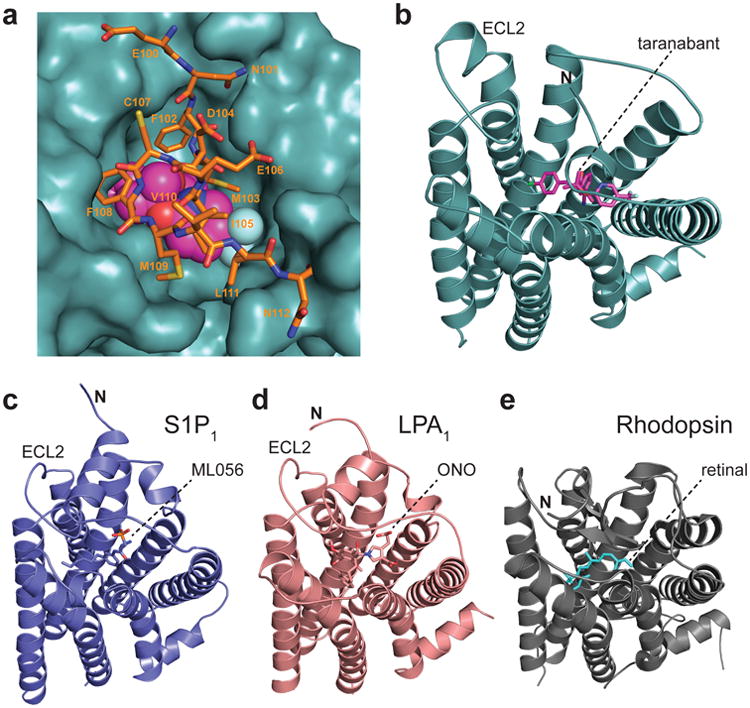
Membrane-proximal N-terminal region of CB1 (a) Interaction between the membrane-proximal N-terminal region and the rest of the receptor. Residues 100-112 are shown as orange sticks, and taranabant is shown as magenta spheres. The remainder of CB1 is depicted as a teal solvent-accessible surface. (b) CB1's extracellular region seen from outside the cell, with the receptor as a teal cartoon and taranabant as magenta sticks. (c) The S1P_1_ receptor (PDB 3v2y) is depicted as a blue cartoon, from the same perspective as in (a) after superposition with CB1. The ML056 antagonist is shown as blue sticks. (d) The LPA1 receptor (PDB 4z35) as a salmon cartoon, from the same perspective as in (a) after superposition with CB1. The ONO 9910539 antagonist is shown as salmon sticks. (e) The GPCR rhodopsin (PDB 1f88) as a gray cartoon, from the same perspective as in (a) after superposition with CB1. The 11-cis-retinal inverse agonist ligand is shown as cyan sticks. Glycosyl moieties in the N-terminal region are removed for clarity.

**Figure 3:**
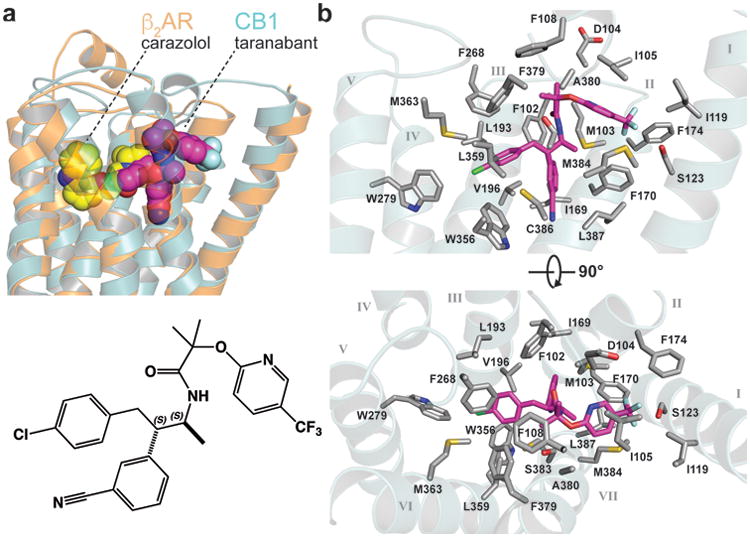
Binding of taranabant to the CB1 receptor (a) Superposition of CB1 (teal transparent cartoon) with β_2_AR (PDB 2rh1, orange transparent cartoon). The rmsd for the Cα positions is 2.6 Å. The beta blocker carazolol is shown as yellow spheres, while taranabant is shown as magenta spheres. At bottom is a 2D representation of taranabant. (b) Contact residues with 4 Å of taranabant in the CB1 structure. The receptor sidechains are shown as gray sticks, the backbone is a transparent cartoon, and taranabant is in magenta. Top view is from the within the plane of the bilayer (TM6 and TM7 cartoon removed for clarity), bottom view is from the extracellular space.

**Figure 4:**
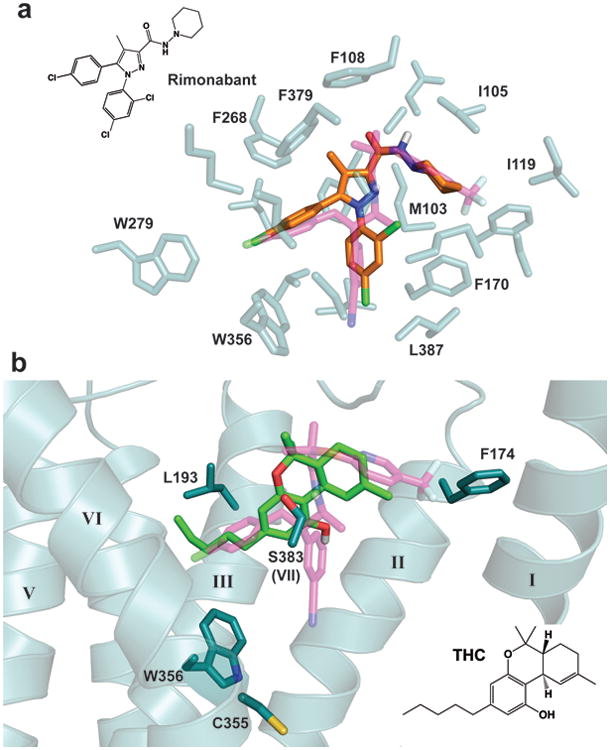
Docking of rimonabant and THC to the CB1 receptor (a) Overlay of the crystal structure pose of taranabant (transparent magenta sticks) with the top-scoring docking pose of rimonabant in orange sticks (see Methods). The contact residues within 4 Å of taranabant are shown as transparent teal sticks. The 2D structure of rimonabant is shown at upper left. (b) Top-scoring docking pose of THC is shown as light green sticks, along with taranabant (transparent magenta sticks). Selected residues important for THC's binding and agonist activity are shown as teal sticks. TM7 cartoon is removed for clarity. The 2D structure of THC is shown at bottom right.
